# Experience sampling methodology in pediatrics: a qualitative analysis of user perspectives on the PROfeel blended mHealth intervention for fatigue

**DOI:** 10.3389/fdgth.2025.1628823

**Published:** 2026-01-02

**Authors:** Anna M. Braspenning, Maartje D. Stutvoet, Sanne L. Nijhof, Elise van de Putte, Remco C. Veltkamp, Rens Brankaert, Inge M. B. Bongers, Eveline J. M. Wouters

**Affiliations:** 1 Tranzo, Tilburg School of Social and Behavioral Sciences, Tilburg University, Tilburg, Netherlands; 2 School of Allied Health Professions, Fontys University of Applied Sciences, Eindhoven, Netherlands; 3 Pediatrics, Wilhelmina Children’s Hospital, University Medical Centre Utrecht, Utrecht University, Utrecht, Netherlands; 4 Information and Computing Sciences, Utrecht University, Utrecht, Netherlands; 5 Systemic Change Group, Department of Industrial Design, Eindhoven University of Technology, Eindhoven, Netherlands

**Keywords:** adolescents, blended care, chronic disease, ecological momentary assessments, experience sampling methodology, implementation, mHealth

## Abstract

**Introduction:**

Children with chronic health conditions are at risk for persistent somatic symptoms such as fatigue, influenced by biological and psychosocial factors. Experience Sampling Methodology (ESM) in mobile Health (mHealth) enables personalized insights into symptom-related factors, potentially improving self-management and quality of life. Blended care—combining digital and in-person support—enhances adherence and interpretation of ESM insights. Despite its potential, no blended ESM intervention has yet transitioned into pediatric care. This study explores direct and indirect user perspectives on the use and implementation of ESM-supported blended mHealth in pediatric care.

**Methods:**

We conducted an exploratory qualitative study on PROfeel, an ESM-supported blended mHealth intervention for fatigue management. PROfeel includes a smartphone-based ESM period, followed by face-to-face feedback and shared decision-making for tailored lifestyle goals. Semi-structured interviews were conducted with patients (*N* = 11), important others (IOs, *N* = 11), and healthcare professionals (HCPs, *N* = 20). Patients, aged 13–25, used PROfeel in a research setting. IOs were primarily parents. HCPs included physicians, nurses, and psychologists.

**Results:**

Patients and IOs identified three themes: (1) wish for improvement vs. ESM effort, (2) value of insights, and (3) support and ownership. Patients were motivated by improvement but found ESM effort challenging. Insights into fatigue and lifestyle factors were valued, though lifestyle change remained limited. Independent use fostered ownership, while parents provided support. HCPs identified the themes (1) balancing patient value and effort, (2) defining roles within the intervention, and (3) concerns about effectiveness, financial coverage, and time. Patient value was the key determinant for PROfeel use, balanced against intervention effort and eligibility criteria. HCPs stressed the need for aligned roles and practical feasibility.

**Discussion:**

ESM-supported care shows promise for children with chronic conditions and may serve as an early intervention. Blended care accommodates autonomy levels and supports ESM insight interpretation. Implementation in hospital-wide care could suit varied clinical needs. Active involvement of end users is essential to maximize patient value and ensure successful integration into pediatric care.

## 1 Introduction

In the Netherlands, approximately 18% of young people between 0 and 25 years has a chronic health condition, like diabetes, juvenile idiopathic arthritis (JIA), asthma, and visual impairments (1). A chronic health condition is defined as a disease that lasts longer than three months, recurs more than three times per year, and/or is linked to long-term medication use, treatments, or aid (1). It can threaten young people's physical, social, and psychological well-being (2) and is a risk factor for developing persistent somatic symptoms, such as pain and fatigue (3–5). Individual differences are seen in the interplay between biological (e.g., sleep, disease activity), psychological (e.g., anxiety) and social (e.g., school pressure) factors associated with these symptoms (6). These differences highlight the need for personalized monitoring, support, and interventions (7).

High-level personalization could be achieved with self-management interventions with the experience sampling method (ESM), also known as ecological momentary assessment (EMA) (8). ESM is an empirically validated structured diary technique embedded in daily life (9). It samples personal experiences and behaviors as well as moment-to-moment changes in well-being via repeated short measurement moments each day, for several days to weeks (10). ESM facilitates self-monitoring, a main component of self-management. This can improve self-insight, support shared decision-making, and promote behavioral change (11). Combining self-management with medical treatment leads to better health outcomes and quality of life for both adults and children (12). Thus, promoting self-management skills is recognized as a key health strategy for managing chronic illnesses (13).

ESM can be offered through mobile eHealth (mHealth). mHealth is particularly well-suited for sustainable support of young people, given their active engagement with the internet and mobile services (14, 15). mHealth's flexibility and adaptability enable personalized approaches across conditions. Moreover, it helps overcome barriers like accessibility, and reduces treatment burden (16). Digital health interventions have been shown to be effective and safe in preventing, detecting and managing mental health issues in youth with chronic health conditions (17). However, despite high expectations, a meta-analysis in 2,410 young patients found only small improvements in self-efficacy and certain disease-related outcomes, underscoring the importance of further research on factors influencing digital treatment effectiveness (18).

Also, integrating ESM in mHealth for young people presents challenges. These include technical hurdles such as survey software problems, logistic issues such as fitting it with school schedules, and preconditions such as the participant's training needs related to developmental abilities. As a result, ESM- compliance rates range from 20% to 95% between studies (19, 20), while sustained therapy adherence is essential for long-term benefits (12).

Combining digital health services with in-person care, known as blended care, could improve treatment adherence (21) and enhance self-management support (13, 22). Self-management support, including help and encouragement from healthcare professionals (HCPs), family members, and close friends, is crucial for boosting an adolescent's ability and confidence in managing their chronic health condition (22). Van Os *et al*. (11) suggested that ESM-supported blended care could facilitate self-monitoring, personalized healthcare, and gaining self-awareness (11). It enables collaborative exploration and interpretation of the ESM-data between patient and HCP (23). This is supported by a recent review on the use of ESM in pediatric care, which found that ESM has the potential to offer personalized feedback to young people with chronic health conditions (19).

Although ESM-supported blended care shows promise, its use in pediatrics remains limited. ESM is still applied mainly for research purposes (19, 24, 25), and the few examples of integrating ESM into blended care interventions have occurred only in research settings (26–30). Since ESM has not yet entered routine pediatric clinical practice, its real-world feasibility in pediatric care remains uncertain. This is further challenged by broader barriers to implementing blended care, such as changes in workflow and technically-challenged staff (31).

Successful implementation in real-world settings depends on stakeholder engagement, a key factor highlighted by implementation models (32). Involving (direct and indirect) users and addressing their specific needs is crucial to fostering the adoption of ESM in pediatric practice. Qualitative research, such as interviews, can provide a nuanced understanding of user experiences and attitudes (33). Therefore, exploring user perspectives on the implementation requirements of ESM is essential for its effective integration into real-world pediatric care.

In summary, the use of ESM-supported blended care is a promising approach to support young people with chronic health conditions and persistent somatic symptoms. However, how to effectively adopt this approach in clinical pediatric practice remains unclear. Therefore, the aim of this study is to examine the direct and indirect user perspectives on the use and implementation of ESM-supported blended care in pediatric practice. To achieve this, we conducted an exploratory qualitative study of PROfeel, an ESM-supported blended mHealth intervention focusing on fatigue, which has so far only been used in research settings (27, 29, 34). In this study, we carried out interviews among direct and indirect users. Direct users were defined as patients and HCPs, indirect users as the important others (IOs) of patients (35, 36).

## 2 Materials and methods

### 2.1 Research design

The study utilized a qualitative research design, employing interviews as data collection method to capture the diverse perspectives from participants regarding the use and implementation of ESM-supported blended mHealth in a pediatric setting. As example, the PROfeel intervention was used (37).

This study adopts an interpretivist epistemological stance, which emphasizes understanding the subjective meanings that individuals (i.e., patients, IOs, HCPs) ascribe to their experiences. From this perspective, reality is seen as socially constructed and context-dependent, shaped by cultural, historical, and interpersonal factors (38). The interpretivist approach aligns with the aim of this study. Rather than seeking universal or objective truths, this study focuses on uncovering patterns of meaning unique to the specific context and individuals studied.

### 2.2 PROfeel intervention

PROfeel has proven its potential for preventive and personalized treatment of young people with a chronic health condition suffering from fatigue. It was shown to be feasible and useful in a feasibility study (27), and effective in reducing fatigue and improving quality of life in both a randomized controlled trial (RCT) (34) and a single-case study (29). These studies were conducted at the Wilhelmina Children's Hospital of the University Medical Centre Utrecht (27, 29, 34), a tertiary care hospital, and the Princess Máxima Center for Pediatric Oncology (27). Both hospitals are located in Utrecht, the Netherlands.

**Figure 1:**
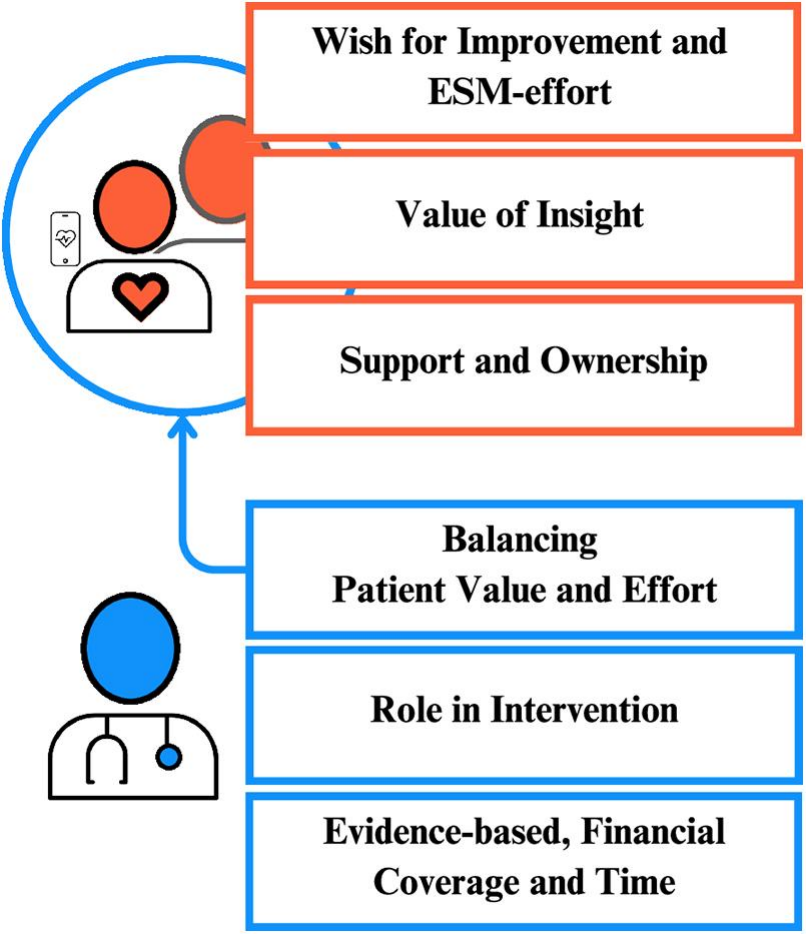
User perspectives on PROfeel, an ESM-supported blended care intervention. Themes from patients and their important others (IOs) are shown together in orange, as their views largely aligned. Themes from healthcare professionals (HCPs) are shown in blue. The three patient/IO themes overlapped with the HCPs' theme “*Balancing Patient Value and Effort*”, as indicated by the blue arrow. Pictograms by Ricardo Orichio from Noun Project (CC BY 3.0).

PROfeel constitutes of four subsequent stages, which have been described more elaborately elsewhere (37). In PROfeel, the face-to-face intervention elements are provided by an HCP called the PROfeel-Practitioner. In short, in stage 1, the ESM-Personalization, the PROfeel-Practitioner introduces the PROfeel app to the patient. In the PROfeel app, the patient personalizes the ESM-questionnaire by choosing relevant items on physical symptoms (e.g., stomach ache), behavior (e.g., hiding emotions), feelings (e.g., feeling enthusiastic) and thinking (e.g., stress about symptoms). The personalized items are added to generic items (e.g., on physical activity level, sleep). In sum, the ESM-questionnaire covers potentially fatigue-perpetuating biological, psychological, social or environmental factors (Supplementary Material S1—Table 1 for ESM-questionnaire items) (37). Examples of items are: “In the last 3 h, I felt fatigued”, “In the last 3 h, I felt happy” and “In the last 3 h, I was physically active”. Items are answered on a visual analogue scale ranging from 0 (“not at all”) to 100 (“very much”, Supplementary Material S1—Figure 1a for example slider). In stage 2, the four-week ESM-Period, the patient fills out five short ESM-questionnaires a day via the PROfeel app. In stage 3, the ESM-Feedback, the patient receives personalized insight into fatigue-associated factors based on the ESM-Period from the PROfeel-Practitioner (Supplementary Material S1—Fig. 1b and 1c for example feedback). This insight is the starting point for shared decision-making on tailored lifestyle advice. In stage 4, the Lifestyle Change Period, the patient works independently on the lifestyle advice. IOs of patients under 16 years of age attended the face-to-face conversations with the PROfeel Practitioner. For patients aged 16 and older, IOs were present only if the patient wished. Beyond this, IOs had no formal role during the PROfeel stages.

### 2.3 Participants and recruitment

Participants in the current study included 1) patients (former users of PROfeel), 2) their IOs, namely parent or partner (indirect former users) and 3) HCPs, who were either former and/or intended future users. They were recruited between September 2022 and March 2023.

#### 2.3.1 Patients and their important others

All the patient participants had experience with PROfeel as participants of the RCT (34). They were young people with (a history of) severe fatigue, and diagnosed with either myalgic encephalomyelitis/chronic fatigue syndrome (ME/CFS), or JIA. To foster diversity in perspectives, no adherence or completion thresholds were applied for inclusion. RCT participants who had given consent to be contacted for future studies were approached via email by the RCT researcher (for all patient groups), or by their treating pediatrician during outpatient clinic visits (for JIA group) after completion of the RCT. A maximum of one reminder email was sent. Patients were asked to identify a person who had supported them during PROfeel and, if applicable, had attended the conversations with the PROfeel Practitioner. This IO (i.e., parent or partner) was contacted with the patient's permission.

#### 2.3.2 Health care professionals

All HCP participants (had) worked in one of two hospitals in which the PROfeel studies had been conducted (27, 29, 34). HCPs had a patient population of adolescents with a chronic health condition, of whom some patients had severe fatigue. Most HCPs had no prior experience with PROfeel. Some had been involved in earlier studies with PROfeel (27, 29, 34). Involvement ranged from patient referral to the PROfeel studies, to designing PROfeel or being PROfeel-Practitioner. We purposively approached HCPs based on a diversity of roles (e.g., pediatrician, psychologist), subspecialties (e.g., oncology, cardiology), and familiarity with PROfeel.

### 2.4 Data collection

#### 2.4.1 Procedure

Participants were informed of the procedure and purpose of the study, and the voluntary nature of their participation. Confidentiality was guaranteed. Informed consent was obtained via signing an informed consent form or via verbally confirming consent on video record. Each patient participant received a 10 euro e-gift card. Interviews took place at the preferred locations of the participants: at home (for patients and IOs), in the hospitals or online via Microsoft Teams (for all participant groups). They lasted approximately one hour. The first four interviews of each participant group were conducted by two investigators (AB and MS), one as interviewer and the other as observer. At the start of each interview, we gave a short recap of the intervention elements of PROfeel, since participants had only limited or no experience (HCPs) or a considerable amount of time had passed since involvement in PROfeel (range patients and IOs: 1–22 months, range HCPs: 0.5–4 years). Interviews were recorded with an audio recorder (offline interviews) or via Microsoft Teams (online interviews).

#### 2.4.2 Interview guide

Demographic variables (age [all], sex [all], ESM compliance and lifestyle rating [patients]) were collected. To explore the perspectives of patients and their IOs on the use of PROfeel, we based the interview guide on Unified Theory of Acceptance and Use of Technology (UTAUT) (39). The same constructs were discussed with patients and IOs, but the exact questions were adapted to the perspective explored (direct or indirect user), and age and level of understanding of the participant. For example, for the construct “performance expectancy”, we asked patients “What did you expect from the app?” and IOs “What were your expectations of the PROfeel treatment?”. To explore the perspectives of HCPs on the use and implementation of PROfeel, we based the interview guide on the Normalization Process Theory (NPT) (40). For example, for the construct “coherence”, we asked HCPs “What goal would you have in mind for using PROfeel in clinical practice?” (Supplementary Material S2—Interview Guide). The interview guide outlined key areas for exploration based on the UTAUT and NPT frameworks, ensuring that all relevant topics were covered. At the same time, it allowed for flexibility, enabling a deeper examination of emerging issues.

### 2.5 Data analysis

Interviews were transcribed verbatim and pseudonymized. Two coders (AB and MS) conducted qualitative thematic coding analysis (38) using the nVivo qualitative analysis software package (41). The first two interviews with HCPs, and the first interview with a patient and an IO (four in total) were independently and inductively open coded by two researchers. After discussion, a common coding strategy was developed. All subsequent interviews were open coded using this strategy. Constant comparison between data and coding, and frequent meetings between AB and MS led to axial and final coding of overarching themes. Data of HCPs was analyzed separately from the data of other participants. In addition, AB and MS independently summarized the transcripts coded by the other to foster content-driven discussion. Data saturation on the overarching code and deeper meaning level was reached for all participant groups.

### 2.6 Reflexivity

The research team members had diverse backgrounds, which resulted in different perspectives on the data. The interview guide was primarily developed by AB (health scientist), and reviewed by MS (medical doctor), EvdP (pediatrician), EW (medical doctor and social scientist) and IB (biomedical scientist). Interviews were conducted by AB and MS. MS was involved in the further development of PROfeel, which might have impacted the data collection and interpretation. This potential impact was minimalized by frequent meetings between AB and MS during the coding and analysis process to discuss key messages. On top of that, the credibility of the analysis was aided by ongoing discussion with two additional reviewers EW and IB, both experienced in qualitative analysis. None of the researchers conducting the interviews and/or analyses had been involved the PROfeel feasibility study or RCT.

## 3 Results

### 3.1 Study population

In total, 42 participants were interviewed. Eleven participants were patients, eleven were IOs, and twenty were HCPs (see Table 1, more details in Supplementary Material S3—Participant Characteristics). Ten patient-parent dyads took part. Patients' age ranged from 13 to 25 years. Nine patients were diagnosed with JIA and two with ME/CFS. Five patients had obtained a good compliance, defined as completing ≥70% of the ESM-questionnaires. Patient ratings of lifestyle advice effectiveness ranged from 2 to 10. One patient withdrew halfway through the RCT, reporting the ESM-Period to be emotionally challenging (see Section 3.2.2). HCPs included pediatricians, nurse (researcher) specialists, and psychologists. The pediatricians had a specialization in immunology, oncology, nephrology, cardiology, pulmonology or social pediatrics. The nurse specialists worked at the day treatment center or were involved in outpatient care for the rheumatology, general pediatrics or social pediatrics department. Of the twenty HCPs, nine had earlier experience with PROfeel, ranging from referral to being PROfeel-Practitioner (see Table 1, Supplementary Material S3—Participant Characteristics). In all stakeholder groups, the majority was female and all participants were white.

**Table 1:** Participant characteristics.

Participant group and corresponding characteristics	Median [IQR] or *N* (%)
Patients (*N* = 11)	
Age^a^ (years)	17,5 [15; 20]
Sex (female)	10 (91%)
ESM-compliance^b^ (%)	62 [44; 89]
ESM-Feedback rating^c^ (0–10)	7 [5; 8]
Important others (*N* = 11)	
Age^a^ (years)	47 [44; 55]
Sex (female)	10 (91%)
*Relationship to patient:*	
Mother	9 (82%)
Father	1 (9%)
Partner	1 (9%)
Health care professionals (*N* = 20)	
Age^a^ (years)	46 [37; 57]
Sex (female)	14 (70%)
*Function:*	
Pediatrician	9 (45%)
Nurse	6 (30%)
Psychologist	5 (25%)
*Experience with PROfeel:*	
Referral of patients	5 (25%)
Research team	4 (20%)
None	11 (55%)

All respondents were white.

^a^ Age is the age during the interview, which can differ from the age of PROfeel experience.

^b^ Patients were informed before the start of the ESM-Period that 70% compliance was sufficient for good quality ESM-Feedback.

^c^ Answer to question: “How much did the personal lifestyle advice help you on a scale from 0 to 10?”.

ESM, experience sampling methodology; yrs, is years.

### 3.2 Themes identified from patients and important others

The views of patients and IOs mostly aligned. We identified the following three themes from their joint perspectives on using PROfeel: 1) wish for improvement and ESM-effort, 2) value of insight, and 3) support and ownership (see Figure 1 for an overview of the themes, Supplementary Material S4 for a more comprehensive overview of supporting quotes).

#### 3.2.1 Wish for improvement and ESM-effort

Patients and their IOs described the substantial negative impact of fatigue on daily life. Some, diagnosed with JIA, perceived it as an inherent aspect of the disease, considering it an unsolvable symptom. Most were motivated to start with and make the required effort for the ESM-intervention PROfeel to better understand their fatigue, how daily activities influenced it, and how they could make changes to improve their well-being.

Patient, 25 years: *“But I did suffer a lot from my fatigue, so I did want very much to do that well in the hope that that would have an effect as well.”*

However, a couple had no or low expectations of the intervention's effect at the start, and many were (partially) motivated by contributing through participation in research. One patient was skeptical given the limited understanding of chronic fatigue.

Patient, 20 years: *“I knew from my doctor that there is still little known about chronic fatigue and how to improve it. […] So I fully participated and followed everything, but I wasn't really thinking that it might actually help.”*

Regarding the required effort for the ESM-Period, almost all participants provided positive feedback, noting that the ESM-questionnaires were short, non-sensitive, quick, and easy to complete on a smartphone. Additionally, the questionnaires were seen as relevant because they were personalized.

Patient, 18 years: *“So it was just, click here, fill it out, and I'm done. It was really easy and quick, so I really liked that.”*

On the other hand, many did not manage to obtain the required ESM-compliance level (Table 1, Supplementary Material S2—Participant Characteristics). A few participants noted that the daily frequency was too high, and the ESM-Period duration too long. Location had a large impact on motivation and feasibility of filling out ESM-questionnaires. Responding in time was especially challenging during school hours, for patients prioritized paying attention to the classes, wanted a private moment, and often were not allowed to use their phone. Responding in time was generally easy when being at home. Four IOs and one patient indicated that gamification of the app (e.g., a reward system, streaks) might sustain motivation.

Parent of 17-year-old: “*[taking perspective of daughter]* '*Then I didn't want to cause a fuss in class with the teacher. I'd just do it quickly during the break*'*, but yeah, she had to do it during the break, and those breaks are really short at school, so then she couldn't chat with her friends.”*

#### 3.2.2 Value of insight

For most patients and IOs, PROfeel's added value was the insight they obtained in fatigue patterns and related lifestyle factors. For a handful, the ESM-feedback was just a confirmation of what they already knew. One dyad remarked that, while the feedback offered no new information at this point, it would have been especially helpful a few years earlier, when they still had limited understanding of the patient's fatigue patterns.

Parent of 15-year-old: *“I think it has given [daughter] a bit more insight into how she can deal with that fatigue. She has also taken out the things that were doable for her. I think that if she hadn't done it, she might still have gone straight to bed right after school.”*

Insight was predominantly obtained with the ESM-Feedback. The preceding ESM-Period led to increased awareness for some. This increased awareness was both perceived as valuable and as confronting. For example, one patient described how daily reflection helped her recognize fatigue patterns. Another found it emotionally challenging to repeatedly report on her ongoing symptoms, making her feel different from peers.

Patient, 16 years: *“Sometimes you don't realize you're tired, but when you look back, you think, ‘Oh, I was actually quite tired that day.’ So, that’s helpful to look for the cause of the fatigue.”*

Patient, 21 years: *“When you have to fill out on your screen multiple times a day that things aren't going well at that moment. You really reflect on it a lot, realizing you're somewhat different on average. It’s a bit confronting.”*

Insight did not automatically lead to lifestyle change. Most patients reported applying the lifestyle advice for a few days or weeks, but few sustained it. Implementation was challenging due to diverse factors such as contextual factors (e.g., friends living far away, COVID lockdown), advice-related factors (e.g., lack of specificity, not fitting their daily life), and personal factors (e.g., limited discipline, expecting little or no effect). Still, most patients and IOs valued the insight, citing recognition of fatigue, greater acceptance, and an improved ability to make conscious choices.

Patient, 25 years: *“Especially when I'm tired, I tend to give myself more leeway, like: ‘I'm so tired, I can take a nap*'*. Whereas if the rules are very strict and clear, you just know: ‘Okay, but if I take a nap now, I'm kind of… I have to actively choose not to follow the rules’.”*

#### 3.2.3 Support and ownership

In most cases, a parent joined the ESM-feedback but had little direct involvement in the ESM- and Lifestyle Change Period. Almost all IOs had a supportive role: often stimulating and reminding during the ESM-Period, sometimes facilitating or joining in the Lifestyle Change Period. Usually, this supportive role was appreciated by both parties. Some parents highlighted the challenges of puberty, where adolescents need support but often resist it due to their growing desire for independence. A couple of patients described taking more control over one's own life with age.

Parent of a 17-year-old: *“Also, to occasionally encourage and motivate her, because she’s right in the middle of puberty, which makes it difficult. She might just think, 'Oh, I'm done with this'."*

Patient, 15 years: *“I can manage independently now, but if I had started a few years earlier, my mom would have had to help me.”*

There was little to no involvement of peers (e.g., friends), because patients mostly considered PROfeel as something private. For instance, one patient filled out the ESM-questionnaires at the school's restroom.

Patient, 25 years: *“Not because I*’*m ashamed or anything …* *but it's still something private, and I don't want to share it with everyone.”*

Both patients and IOs appreciated the parental independence during PROfeel, reinforcing patient ownership. A few emphasized that the ESM-Feedback should primarily involve the patient rather than the IO to strengthen this sense of ownership. Additionally, many appreciated that the lifestyle advice resulted from shared decision-making, further supporting patient ownership.

Parent of 15-years-old: *“So that was really good, the space that was given to [daughter] to respond herself. [taking perspective of daughter] ‘I'm not here for nothing, I'm not just a patient. I also get to have a say.’ And that is really important, even if they're only 14.”*

### 3.3 Themes identified from health care professionals

HCPs described fatigue as a significant burden for patients, as its cause was often unclear, making it an elusive symptom for which few effective interventions exist. Consequently, HCPs welcomed PROfeel as an intervention. We found the following three themes illustrating the HCPs' perspectives on the use and implementation of PROfeel: 1) balance between patient value and effort, 2) role in intervention 3) evidence-based, financial coverage and time (see Figure 1 for an overview of the themes and Supplementary Material S4 for a more comprehensive overview of supporting quotes).

#### 3.3.1 Balance between patient value and effort

The importance of the intervention's value for the patient was central in HCPs' discussions. They spoke about the expected effect, the effort required from patients in the ESM- and Lifestyle Change Period, and the resulting patient eligibility criteria. HCPs reported observing patients suffering from persistent fatigue and noted the challenges associated with treating it. They expressed a strong desire for a low-threshold treatment option to offer as a solution for managing fatigue. HCPs thought the ESM-intervention PROfeel could help patients gain insight into their fatigue and daily living patterns, make lifestyle adjustments based on practical advice, and possibly improve their ability to manage or reduce fatigue.

Pediatrician: *“But that is, of course, incredibly insightful. Because you*’*re going to discover patterns that the patient didn*’*t even know existed. So, naturally, you*’*re going to provide the patient with insight into where the fatigue comes from and what helps to address it.”*

HCPs shared concerns about the potential intrusiveness of the intense ESM-Period in daily life. Some also expressed apprehension that emphasizing fatigue during the ESM-period could exacerbate symptoms.

Psychologist: “*Well, children, aren*’*t going to fill it in five times a day. They just won*’*t do it, and then there*’*s also the question of whether it*’*s even healthy to log your symptoms five times a day.”*

They anticipated that the effort required from patients to use PROfeel, combined with the absence of immediate effects from the intervention, might cause motivational challenges.

As a result, HCPs felt that only highly motivated patients would be suited. To motivate patients, some HCPs emphasized the importance of clearly explaining the added value for the patient during the introduction of PROfeel.

Nurse researcher: “*A strong intrinsic motivation from the patient is necessary; without it, they won*’*t complete the diaries, and you won*’*t get a reliable profile. In that sense, it depends on patients experiencing enough issues to want to take action themselves. If someone has that intrinsic motivation, I think you can indeed provide a very valuable and personalized piece of advice.”*

Opinions diverged on which patients would benefit most. As for the duration and severity of fatigue, one HCP stressed that PROfeel should be used as an early intervention. It could prevent patients from becoming stuck in non-contributing patterns which are harder to change. By others, the ESM-Period was also seen as a general diagnostic tool, that could be used for a broad range of fatigue severity. In the case of mild symptoms, the intervention as a whole would be suited, while in the case of severely debilitating symptoms, the ESM-Period and -Feedback could serve as a stepping stone toward more intensive support.

Pediatrician: *“That every patient with persistent physical symptoms should first undergo PROfeel because it also provides insight into the correlation between thinking, feeling, and behavior, which is also very useful for the start of more intensive support.”*

Furthermore, sense of agency over health was seen by one HCP as a prerequisite for a successful intervention. Others, however, believed that PROfeel could play a role in fostering this agency, helping patients take more control over their health.

Psychologist: *“The most important thing is to gain more self-insight, and therefore also more awareness that there are things the patient can do to reduce symptoms. I don*’*t think lifestyle advice necessarily solves this, but it gives the patient the experience that to a certain extent, they have control over their symptoms.”*

Concerning age, older adolescents were considered more capable of working independently on PROfeel, while at the same time requiring more support due to being in puberty. Additionally, adjustments were suggested for the graphs used in the ESM-Feedback (e.g., simplifying visuals for younger users), the wordings used in the ESM-questionnaires and app style.

Pediatrician: *“We noticed that the report was quite challenging for some of the younger children, so you need more textual explanations of what we*’*re seeing here before you can reflect on it together, while the older children could interpret it more easily themselves.”*

#### 3.3.2 Role in intervention

HCPs held diverse views on their responsibilities and subsequent roles, although all agreed that these roles needed to be clear to adopt PROfeel effectively. HCPs agreed that the pediatrician was the preferred HCP to identify patients suited for PROfeel by screening for fatigue and ruling out direct treatable medical causes.

Pediatrician: “*I would start with anyone who has an abnormal fatigue score, but you need to verify that first. You need to check whether they are indeed more fatigued than normal, and then you can discuss: ‘Hey, we might have a treatment for that. Something you could try’.”*

Although most HCPs preferred another type of HCP than the pediatrician to take an active role as the PROfeel Practitioner in the blended care, their reasons varied. Some felt that fatigue was outside their scope of responsibility, focusing only on the disease (e.g., the heart defect or cancer), excluding fatigue that could not be directly medically explained. Even those who took a more integrative approach, considering the patient's overall well-being, including persistent fatigue, still preferred another HCP over the pediatrician. Reasons given were the required skills, time constraints, and alignment with existing care paths. The required skills included understanding the ESM-Feedback, and communication techniques to motivate patients for lifestyle change.

Specialized nurse: “*Medical specialists don*’*t always feel responsible for everything, and I can understand that. Naturally, they look at the whole child, but their main focus is still on the illness. That balance can sometimes be a bit challenging, so occasionally things come up that they don*’*t always feel they have the space for or that they feel is part of their responsibility.”*

Nurse researcher: “*But you also want someone who is capable of providing these recommendations effectively. So, it’s a matter of figuring out what skills someone needs to be able to deliver this advice and determining who is the most available for the role.”*

Whilst some HCPs (pediatricians and nurse specialists) saw their communication techniques as sufficient, others (pediatricians and psychologists) expressed the need for extra training if the PROfeel-Practitioner was not a psychologist.

Pediatrician: *“I think that person is either a psychologist or a pedagogue, someone who is familiar with this method. A psychologist would have an advantage because they understand more about the relationship between thoughts, feelings, and behaviors. […], as long as they fill the gap between somatic medicine and lifestyle.”*

HCPs agreed that PROfeel should be seamlessly integrated into existing care pathways without altering them. This would enhance the chances of adoption. However, preferences for integration varied, reflecting differences in care paths across subspecialties and patient groups. This variation extends to factors such as frequency, type and goal of patient visits, as well as the types of HCPs involved, ranging from only a pediatrician to a multidisciplinary care team.

Nurse researcher: “*Every department has its strengths and challenges, which present certain obstacles, and an existing structure cannot simply be adopted by every outpatient clinic. Therefore, it is not a universal solution to implement it more broadly in the same way. For example, for some patient groups, it may not be desirable to have them visit the outpatient clinic at regular intervals and to always involve a multidisciplinary team in the process.”*

Regarding the implementation of PROfeel in clinical practice, HCPs indicated that a dedicated HCP within their subspecialty would be helpful. This HCP could promote PROfeel to direct colleagues by sharing positive experiences of patients with PROfeel, showing its value.

Pediatrician: *“It's generally very well known that people tend to experience overwhelm with new things, and I already have so many lists and tasks to complete. What's necessary is to have a few good examples where something really works and where you demonstrate, especially, that it doesn't take much time but it does yield results.”*

#### 3.3.3 Evidence-based, financial coverage and time

Scientific evidence for the effectiveness of PROfeel was considered essential by HCPs. Some HCPs pointed out that if the effectiveness of PROfeel was shown in patients with another disease, assessing its effect in their own patient group would increase the likelihood of continued use of PROfeel. A second precondition was financial coverage for both the mHealth and blended care component. A few linked this to cost-effectiveness, explaining that this was needed for healthcare insurers to provide coverage. A third precondition was time, both for conducting and implementing PROfeel. Most HCPs expressed concerns about lacking the time to dedicate more to patients, which would be necessary if they were to take on the PROfeel Practitioner role on top of their current responsibilities. One pediatrician stressed that the added patient value prevailed over the extra time it would cost. Some did not perceive time as a barrier, as they thought the time investment would be minimal.

Pediatrician: *“No, we just don*’*t have time for that, and neither do general pediatricians. So if you want to incorporate [PROfeel], you just have to really limit it tightly.”*

Pediatrician: *“And if that means we need someone to invest three hours a week because that helps while other things don*’*t, well then, that's how it is, right? We're striving for healthy, independent, and happy young people after all, so sometimes you have to invest in that.”*

## 4 Discussion

### 4.1 Principal results

This study examined the use and implementation of ESM-supported blended mHealth in pediatrics by exploring the perspectives of direct and indirect users on the PROfeel intervention for youth with chronic conditions and persistent fatigue. From interviews with patients and their IOs, we learned that PROfeel's value for patients was determined by their wish for improvement, the required effort for the ESM-intervention, the resulting insight into fatigue and associated lifestyle factors, and the balance between support and ownership. From interviews with HCPs, we learned that value for the patient was the central determinant for their use of PROfeel. This patient value was counterbalanced by the patient's effort required, and resulted in patient eligibility criteria. Other important factors were clearly defined roles within the intervention that aligned with responsibilities, skills and existing care paths, as well as the preconditions effectiveness, financial coverage, and time.

### 4.2 Integrated perspectives of patients, important others and health care professionals

HCPs' views on value for the patient can be linked to the themes found for patients and IOs (Figure 1). Therefore, in the following section, we will focus on patient value from the perspectives of patients themselves and their IOs, and of HCPs. Our main finding was that HPCs emphasized patient value for the adoption of PROfeel, showing the importance of patients in the implementation process, even though their direct influence may be limited (32).

#### 4.2.1 Motivation and the required effort

The motivation for PROfeel among patients stemmed from a desire to understand and improve fatigue, and its level corresponded to their performance expectancy (39). Most patient participants were diagnosed with JIA, a disease in which fatigue is a prevalent yet complex symptom, with an unclear etiology (42). Patients may appreciate the intervention, because it acknowledges and addresses their persistent fatigue (43). Additionally, the lack of alternative low-threshold treatments likely contributes to the high motivation expressed by patients and HCPs. HCPs saw a need for high patient motivation, given the required ESM- and lifestyle change efforts for patients. In other words, from their perspective, high performance expectancy could compensate for high effort expectancy (39). Low ESM-compliance, observed in some of our patient participants and in other ESM studies in adolescents (20, 44), compromises the validity of ESM-results (45), and consequently the intervention's effectiveness. Our results on ESM-effort support recommendations of previous studies that the feasibility of the ESM-Period in adolescents may be improved by reducing its intensity and duration, aligning the ESM schedule with school schedules, providing game incentives, and offering support from a close adult (20). Although many eHealth apps targeting youth aim to foster engagement (46), this engagement is often short-lived. Common reasons include game elements that are poorly integrated with the app's primary purpose or insufficiently adaptive to the user's needs (47). In the development of future ESM-interventions, repeated feedback sessions with patients could ensure better alignment with patient preferences and enable iterative improvements to the intervention (48). Such alignment not only benefits the patient directly but also indirectly enhances acceptance and engagement among HCPs by increasing patient value, thereby facilitating broader implementation.

#### 4.2.2 Balancing support and ownership

Participants discussed how PROfeel facilitates patient ownership, partly by the independence from parents in its use, while also emphasizing the importance of parental support. This apparent contradiction highlights the challenge adolescents with chronic health conditions and their parents face as they transition from parental management to self-management (49), a shift that aligns with the development of autonomy during adolescence (50). The shared decision-making during the ESM-Feedback on the lifestyle advice is an example of how HCPs may support the patient-parent dyad in finding the right balance between patient autonomy and the individual needs of each child (49, 51, 52), as it is well established that HCP involvement can support patients in achieving personal autonomy (52).

While friendships typically gain significance during adolescence (49), patients reported little involvement of friends in PROfeel. This aligns with previous research on care preferences of adolescents with chronic health conditions, showing minimal peer engagement in self-management (53). This may be related to wanting to live a “*normal life without prejudice*”, as explained by children with JIA in a qualitative study (54). This “*normal life*” can be facilitated by use of mHealth in ESM-interventions for youth, as filling out the ESM-questionnaires on a personal smartphone renders no overt differentiation or stigmatization. Nevertheless, the current trend of banning smartphones from schools (55) may present a significant obstacle for children who don't want to be the exception for being allowed to use their phones during school hours.

#### 4.2.3 Insight into fatigue and associated lifestyle factors

The majority of patients, IOs and HCPs highly valued the insights the patient gained through the ESM-Period and the ESM-Feedback. However, variability in insight—and thus perceived value—may relate to several factors. These include how well the ESM-items captured physical symptoms, behaviors, feelings, and thoughts relevant to each patient. Although patients could personalize items, choices were limited to predefined options, with only one fully open-ended item. Notably, no participants reported dissatisfaction with item selection. Additionally, the types of fatigue associations identified in the ESM-Feedback (e.g., with daytime resting vs. being sad) may influence how easily insights translate into actionable lifestyle advice. Future research using a quantitative design could explore the relationships between personalization choices, association types, and user value.

The wish for insight into symptoms was also seen in the study of Lalloo *et al.* (56), in which adolescents with chronic pain frequently utilized the voluntary symptom tracking functionality of the app (56). That ESM can result in personal insight into the context specificity of symptoms has been reported in adults with chronic cancer-related fatigue (57) and psychiatric diseases (58, 59), as well as earlier adolescent users of PROfeel (27). Such insight might foster resilience by reducing uncertainty and increasing active coping (60). It is considered the main strength of ESM (61). However, in our study, some participants also expressed concerns about the negative impact of symptom focusing due to ESM. This concern could hinder the implementation of ESM-based interventions. The discrepancy in ESM-experiences, in which the majority profits, but a small proportion of patients reports negative effects, has also been observed by Bos *et al*. (58). Patients might be capable of accurately assessing beforehand whether they will experience ESM as positive or negative, based on the earlier PROfeel study of Nap- van der Vlist *et al*. (27). One of the main reasons for the 97 patients approached not wanting to participate (41%) in Nap- van der Vlist's study was not wanting to be confronted with their fatigue, while only 5% (*N* = 3) dropped out for this reason (27). To minimize the potential negative effects of the ESM-Period, the involved HCP should be aware of its potential negative consequences (58), and offer the option for early termination of the ESM-Period if needed. Regarding the ESM-Feedback, blended care ensures HCP guidance in the patient's interpretation of findings and subsequent actions (57). For a more scalable, fully digital ESM-intervention, the value and safety of automated personalized feedback without HCP involvement needs to be explored.

Opinions varied on the optimal timing for PROfeel for achieving the greatest benefit. Some suggested it as an intervention for patients experiencing severe and disabling persistent fatigue. The effect of PROfeel on diminishing fatigue in this group has already been shown (29, 34). Others preferred PROfeel as an early intervention, when persistent fatigue was present but did not hinder daily life (yet). The PROfeel single case study showed that participants with relatively milder symptoms profited most (29). Additionally, research in adolescents with subclinical depression has demonstrated that ESM-self-monitoring can alleviate symptoms by raising awareness (62). These findings highlight the potential of ESM-based insights as an early intervention. Moreover, they suggest that ESM may function as an intervention in itself. This raises an important question: is PROfeel's effectiveness driven primarily by the ESM-Period, or by its combination with ESM-Feedback and meaningful lifestyle changes? Future research should explore PROfeel's value as an early intervention. A single-case design including patients with varying fatigue severity could help identify when PROfeel is most effective and which components are essential to its effect.

### 4.3 Implementation factors beyond patient value

The focus of patients and their IOs was on their individual experiences. While HCPs emphasized the value for individual patients, they also provided broader perspectives on barriers and facilitators for a blended mHealth ESM-intervention like PROfeel, particularly regarding roles and preconditions.

#### 4.3.1 Scope of responsibilities and heterogeneity of practice

Although identification of eligible patients was seen as a responsibility for the pediatrician, HCPs were challenged in determining who should fulfil the role of the PROfeel-Practitioner in the blended care process. Pediatricians were reluctant as the integrative approach required extends beyond their specialized care areas. While most acknowledged the importance of addressing non-specific symptoms like fatigue, many lacked familiarity and training in this domain (63). This reluctance may be reinforced by the complexity of tertiary care settings, which often demand a narrower, disease-specific focus (64). Moreover, fully integrating PROfeel into the heterogeneous disease-specific care paths may not be feasible or desirable, as it would require tailoring PROfeel to each care path or changing existing workflows. Instead, for interventions like PROfeel in tertiary care settings that are not specific to a single disease, we suggest a hospital-central care path with a dedicated HCP to whom specialized pediatricians can refer. This strategy avoids broadening the specialized focus and altering disease-specific care paths, while safeguarding the expertise and skills required for the ESM-intervention. This approach could enhance the effectiveness and acceptance of such integrative interventions within specialized care environments. The implementation of PROfeel in less specialized contexts, like general hospitals or even non-health care settings like schools, was beyond the scope of this study but remains an area for future consideration. Comparative studies across different care settings could provide valuable insights into the transferability of findings and the contextual factors influencing the use and implementation of ESM-interventions.

#### 4.3.2 Addressing barriers to implementing blended mHealth interventions in tertiary pediatric care

Despite the potential of blended mHealth interventions in pediatric care, several systemic legal, ethical, financial, and technological challenges remain (65). The main systemic barriers identified by HCPs in our study closely resembled those reported in the context of pain monitoring in pediatric oncology. These included difficulties in securing adequate financing, allocating sufficient time, and demonstrating robust evidence for effectiveness. Among these, lack of financial recourses and time allocation were identified as the predominant barriers (66). These challenges highlight the necessity of prioritizing mHealth innovations within healthcare budgets, which are frequently constrained by competing demands.

This paper contributes to the discourse by emphasizing the need for collaborative, multi-level approaches to address these implementation challenges. Effective solutions require engagement from key stakeholders, such as researchers, healthcare institutions, policymakers, and insurers, to develop sustainable strategies. Implementation frameworks like RE-AIM and NASSS illustrate the importance of aligning resources and expertise across the healthcare system to overcome these barriers (67, 68). By integrating these strategies, the adoption of blended mHealth interventions can be accelerated, ensuring long-term benefits for pediatric care practice.

#### 4.3.3 Strengths and limitations

Our study has several strengths. First, data collection within multiple stakeholder groups provided a comprehensive understanding of implementing blended mHealth interventions using ESM in tertiary care settings. Second, the stakeholder groups were diverse in characteristics like age and ESM-compliance for patients, and level of experience with PROfeel and role for HCPs. This diversity allows for a more nuanced understanding of the factors influencing the adoption of mHealth interventions in clinical practice. Third, the multidisciplinary research team with different levels of involvement with the PROfeel intervention itself, ensured a thorough analysis and fair interpretation of the data. Fourth, by conducting implementation research in an early stage, the findings can be used for the further development of the PROfeel intervention as well as the development of an implementation strategy.

Although these strengths enhance the breadth and depth of our study, potential limitations concerning the perspectives captured should be acknowledged. First, our patient respondents were predominantly female, aged 13 and older, and mostly diagnosed with JIA. While fatigue is most prevalent among female adolescents (69) and shows similar patterns across diseases (70), this sample composition may have constrained the diversity of perspectives. Moreover, future studies could further enrich our findings by including individuals from more diverse ethnic backgrounds and analyzing additional data on app interaction, such as screen time. Second, one consideration is that professionals with managerial, legal, ethical, financial, or technological expertise were not included as participants. Their perspectives could offer valuable information about how ESM-interventions function within healthcare organizations, and how preconditions are met. The current study focused specifically on direct and indirect users of an ESM-tool in the Netherlands; therefore, it does not provide a complete overview of the systemic challenges involved, nationally or globally. Third, the variation in organization and delivery of care across different healthcare settings makes it difficult to transfer results obtained from tertiary to secondary or primary care settings. Fourth, as this study was deliberately conducted before PROfeel was implemented in clinical practice, most HCPs had no direct experience, patients and IOs participated in a research setting, and recall varied due to differing time intervals since participation (37). These factors may have influenced the findings.

## 5 Conclusions

In conclusion, ESM-supported blended mHealth is promising for children with chronic health conditions and persistent somatic symptoms like fatigue. The use of mHealth suits the adolescent population because it fosters patient ownership and supports non-stigmatizing use. Blending mHealth with HCP guidance provides an opportunity to tailor interventions to the level of autonomy of individual patients, and helps interpreting ESM-Feedback. The ESM-Feedback has the potential to prevent the progression of symptoms when applied as an early intervention. Providing the intervention through a hospital-wide pathway might facilitate easier adoption over implementation in disease-specific care pathways, and also ensures HCP expertise in the ESM-intervention. Actively involving end users in future development and implementation phases is key to maximize value for the patient and subsequently bridge the gap between research and clinical practice.

## Data Availability

The original contributions presented in the study are included in the article/Supplementary Material, further inquiries can be directed to the corresponding author.
