# Supplementary material for: Experience sampling methodology in pediatrics: a qualitative analysis of user perspectives on the PROfeel blended mHealth intervention for fatigue
Source: Front Digit Health. 2026 Jan 2;7:1628823. doi: 10.3389/fdgth.2025.1628823 (PMC12807976; doi:10.3389/fdgth.2025.1628823)
Supplement: Supplementary file 1 [file Supplementaryfile1.pdf]

Supplementary Material 1 - PROfeel

Table 1: ESM-questionnaire items

Participants receive their personalised ESM-questionnaire five times daily during the ESM-Period. The two sleep items are only posed in the first survey of the day. The participant personalises the questionnaire by doing the following: 1) add up to two extra items on specific physical symptoms 2) choose the exact phrasing of items from a list, and 3) add up to one extra item (not shown in table). Total survey length ranges from 19 (first of the day, 3 extra items) to 14. Examples of personalisation options are displayed between [brackets].

Construct (number of items)	Item	Answer scale
Night rest<br>(2, once a day)	• Last night, I slept	Multiple choice: [0-2 hours] to [more than 12 hours]
	• I felt rested upon waking up	VAS: 0 (not at all) to 100 (very much)
Symptoms<br>(3-5)	• In the past 3 hours, my symptoms were	VAS: 0 (not severe at all) - 100 (very severe)
	• In the past 3 hours, I felt hindered by symptoms in doing the things I wanted to do (e.g., going to school, exercising, seeing friends)	VAS: 0 (not at all) to 100 (very much)
	• In the past 3 hours, I felt tired	VAS: 0 (not at all) to 100 (very much)
	• In the past 3 hours I had [a headache]<br>Examples of other options: back pain/ nausea/dizziness/concentration problems/memory problems	VAS: 0 (not at all) to 100 (very much)
Behaviour<br>(4)	• In the past 3 hours, I [withdrew from my social environment]<br>Examples of other options: avoided activities to prevent symptoms/ignored symptoms/suppressed my emotions/felt bored	VAS: 0 (not at all) to 100 (very much)
	• The last 3 hours I was physically active	
	• In the past 3 hours, I have been lying down/resting/sleeping during the day	
	• In the past 3 hours, I was mentally active (like studying for school)	
Thoughts<br>(1)	• In the past 3 hours, I [had stress from wanting or having to do too much]	VAS: 0 (not at all) to 100 (very much)

	Examples of other options: worried about my symptoms/worried in general/was occupied with what others think of me.	
Feelings (2)	• In the past 3 hours, I felt [happy]<br>Examples of other options: satisfied/loved/proud<br>• In the past 3 hours, I felt [angry]<br>Examples of other options: nervous/anxious/tense/sad	VAS: 0 (not at all) to 100 (very much)
Social context (4)	• In the past 3 hours, I was mostly	Multiple choice<br>■ At home<br>■ At school or work<br>■ With friends or family<br>■ Somewhere else
	• In the past 3 hours, I was mostly	Multiple choice<br>■ Alone<br>■ With others online<br>■ With others offline
	• For the last three hours, I was in touch with people who helped me, supported me and understood me	Yes/no
	• For the last three hours, I was in touch with people who bothered me, who I was in conflict with or who did not understand me	Yes/no

Abbreviation: VAS is visual analogue scale.

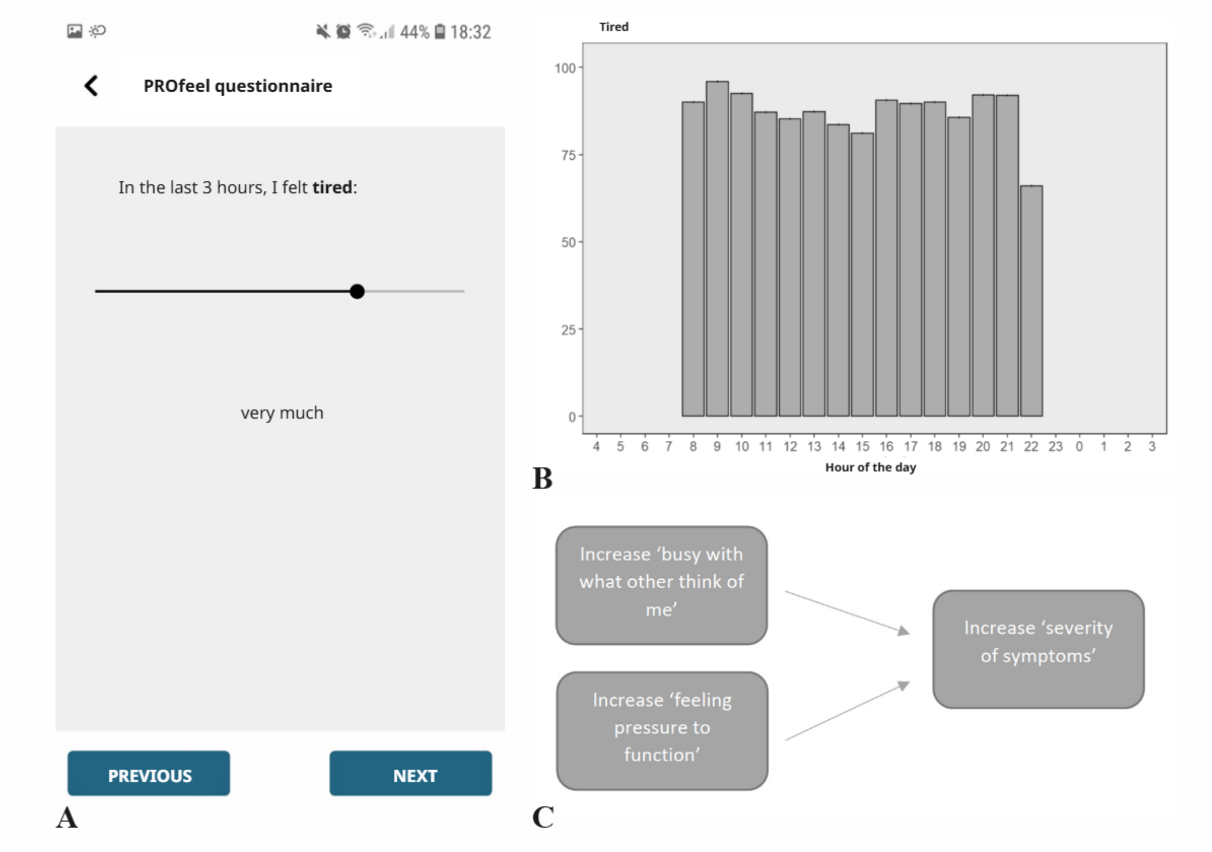

Figure 1: PROfeel app and ESM-Feedback. A) example ESM question with slider. B) and C) example figures from ESM Feedback.

B and C were originally published in: Vroegindeweij, A., Swart, J.F., Houtveen, J. *et al.* Identifying disrupted biological factors and patient-tailored interventions for chronic fatigue in adolescents and young adults with Q-Fever Fatigue Syndrome, Chronic Fatigue Syndrome and Juvenile Idiopathic Arthritis (QFS-study): study protocol for a randomized controlled trial with single-subject experimental case series design. *Trials* **23**, 683 (2022). https://doi.org/10.1186/s13063-022-06620-2
