# Supplementary material for: Experience sampling methodology in pediatrics: a qualitative analysis of user perspectives on the PROfeel blended mHealth intervention for fatigue
Source: Front Digit Health. 2026 Jan 2;7:1628823. doi: 10.3389/fdgth.2025.1628823 (PMC12807976; doi:10.3389/fdgth.2025.1628823)
Supplement: Supplementary file 2 [file Supplementaryfile2.pdf]

Supplementary Material 2 - Interview Guide

1 Interview guide

Interview guide per respondent group
A: Patients
B: Important others
C: Health Care Professionals

2 A: Patients

0. Introductory questions for patients (like: 'What do you like to do in your free time?')
0. What do you use your phone for?
0. When did you use PROfeel?

1. How do you look back on using PROfeel (the app and the conversations)?
2. How did you come to participate in the fatigue research?
3. What did you expect from the app?
4. What was it like to start working with the PROfeel app and lifestyle advice?
5. What was the initial conversation with researcher before starting the diary period like?
6. What was it like to fill out the questionnaires in the PROfeel app?
7. What was important for you to be able to fill out the questionnaires on time?
8. What influence did filling out the questionnaires have on the things you did during the day?
9. What was the conversation with the researcher about the lifestyle report like?
10. What was it like to start working with the lifestyle advice?
11. What did you tell others about starting with the app and the subsequent lifestyle advice?
12. What did PROfeel mean to you?
13. If you could go back in time, what approach would you take to using the app and working on the lifestyle advice?
14. If you could go back in time, would you do the PROfeel treatment again?
15. If you meet a peer who is very tired, what would you tell them about PROfeel?
16. Do you have a tip that would have made the app and the conversations more helpful for you?

3 B: Important Others

Important Others of patients > 16 years old:
0. We are speaking with you because you are important to someone who has undergone the PROfeel treatment for fatigue symptoms. What is your relationship to that person?

All Important Others:
0. Can you describe a typical day in the life of your child/loved one?
0. To what extent are you familiar with eHealth?
0. How did your child/loved one come into contact with PROfeel?
0. When did your child/loved one work with PROfeel?
0. What was your role in your child/loved one's PROfeel treatment?
0. When we talk about the PROfeel treatment, what comes to mind for you?

Parents of patients < 16 years old:
1a. Why did you decide, along with your child/loved one, that he/she would participate in the fatigue research?
Important Others of patients > 16 years old:
1b. What did you think about your loved one participating in the fatigue research?

All Important Others:

2. What were your expectations of the PROfeel treatment?
3. In your opinion, what is the goal of using PROfeel for your child/loved one?
4. What was it like for your child/loved one to start using the PROfeel app and lifestyle advice approach?
5. What was it like /What have you heard about the initial conversation with researcher (..) before starting the diary period?
6. What was it like for your child/loved one to fill out the questionnaires?
7. What was important for your child/loved one in being able to fill out questionnaires on time?
8. What influence did filling out the diaries have on the activities you and your child/loved one did in a day?
9. What was the conversation with researcher about the lifestyle report like?
10. What was it like for your child/loved one to start working with the lifestyle advice?
11. What did your child/loved one or you tell others about him/her starting to use the app and lifestyle advice?
12. What has PROfeel meant for your child/loved one and for you?
13. If you could go back in time, how would you as a loved one see your role in using the app and working on lifestyle advice?
14. To what extent has PROfeel influenced the care your child/loved one receives from healthcare professionals or healthcare institutions?

Parents of Patients < 16 years old:

15. If you could go back in time, would you choose the PROfeel treatment again for your child?

All Important Others:

16. If you were to encounter a (parent of a) peer of your loved one who is also experiencing a lot of fatigue, what would you tell them about PROfeel?
17. In what way would you like the effect of PROfeel for your loved one to be assessed?
18. Do you have any suggestions that would ensure the app and the conversations would have better helped your child/loved one?

4 C: Health Care Professionals

0. What is your current position?
0. What is your role in the care provision for chronically ill children?
0. How many years of work experience do you have?
0. What is your age?
0. Do you have experience with applying eHealth in your patient care?

0. What conditions do the children you see in outpatient care have?
0. How do you experience the care for patients with persistent fatigue symptoms?
0. What experience do you have with PROfeel?
OR
0. What do you already know about PROfeel?

Depending on the healthcare professionals' familiarity with PROfeel, background information will be provided based on a patient journey.

1. What experiences do you have with using PROfeel in a study context?
2. What goal would you have in mind for using PROfeel in clinical practice?
3. In what way should PROfeel be implemented in clinical practice for patients with persistent fatigue?
4. What is needed to introduce PROfeel properly to patients based on those opportunities/ your experiences?
5. What are your preconditions for actually working with PROfeel in practice?
6. What could influence your intention to use PROfeel?
7. How could the use of PROfeel change the care provided by the hospital?
8. What impact could the use of PROfeel have on your daily tasks and responsibilities?
9. Do you have any tips that would make the app more usable?
10. What preparation is needed to successfully implement such a form of blended care in clinical practice?
11. What is needed to sustainably implement blended care?
12. To what extent should the use of blended care be evaluated
