# Supplementary material for: Experience sampling methodology in pediatrics: a qualitative analysis of user perspectives on the PROfeel blended mHealth intervention for fatigue
Source: Front Digit Health. 2026 Jan 2;7:1628823. doi: 10.3389/fdgth.2025.1628823 (PMC12807976; doi:10.3389/fdgth.2025.1628823)
Supplement: Supplementary file 3 [file Supplementaryfile3.pdf]

Supplementary Material 3 – Participant Characteristics

Table 1. Patient's and Important Other's Characteristics.[a-e]

Patient	Age[c] (yrs)	Sex	ESM compliance (%) [d]	ESM-Feedback rating[e]	Time since end PROfeel	IO	Relationship to Patient	Age (yrs)
**P01**	13	M	44	8	5	I01	Mother	38
**P02**	15	F	54	7.5	3	I02	Mother	55
**P03**	20	F	89	3	10	I03	Mother	47
**P04**	21	F	40	5	1	I04	Mother	54
**P05**	17	F	61	7	13	I05	Mother	46
**P06**	20	F	77	3	15	I06	Mother	49
**P07**	15	F	35	8	1	I07	Father	70
**P08**	17	F	98	2	11	I08	Mother	47
**P09[a]**	25	F	99	NA	22	NA	NA	NA
**P10**	16	F	41	8	2	I10	Mother	44
**P11[b]**	21	F	63	10	20	I11	Partner (F)	22
**P12**	18	F	95	6.5	9	I12	Mother	56

[a]P09 did not identify an important other.
[b]P11 did not participate in an interview.
[c] Age is the age during the interview, which can differ from the age of PROfeel experience.
[d]Patients were informed before the start of the ESM Period that 70% compliance was sufficient for good quality ESM-Feedback.
[e]Answer to question: how much did the personal lifestyle advice help you on a scale from 0 to10?
Abbreviations: ESM is experience sampling methodology, F is female, IO is important other, NA is not applicable, M is male, yrs is years.

Table 2. Health Care Professional's Characteristics.[a]

HCP	Function	Sex	Age (yrs)	Experience with PROfeel[a]
**H01**	Medical doctor	F	30	Feasibility study: research team
**H02**	Pediatrician	F	51	Attended stage 3
**H03**	Pediatrician	M	49	None
**H04**	Pediatrician	M	66	Referral
**H05**	Health Researcher	F	33	None
**H06**	Pediatrician	F	65	Research team
**H07**	Specialized nurse	F	62	None
**H08**	Pediatrician	F	42	None
**H09**	Pediatrician	M	37	None
**H10**	Specialized nurse	F	42	None
**H11**	Specialized nurse	F	42	None
**H12**	Pediatrician	M	41	None
**H13**	Specialized nurse	F	31	None
**H14**	Pediatrician	M	45	None
**H15**	Pediatrician	M	57	Feasibility study: referral
**H16**	Pediatrician	F	47	Feasibility study: referral
**H17**	Psychologist	F	26	RCT: research team
**H18**	Psychologist	F	62	None
**H19**	Psychologist	F	51	Feasibility study: attended stage 3
**H20**	Psychologist	F	58	Feasibility study: research team

[a]Time passed since PROfeel experience ranged from 0.5 to 4 years ago. If no study is specified, described experience relates to both PROfeel studies (29,30).
Abbreviations: F is female, HCP is health care professional, M is male, RCT is randomized controlled trial, yrs is years.
